# Anterior ST‐Segment Elevation From Nondominant Right Coronary Artery Occlusion: Lessons From Two Cases

**DOI:** 10.1155/cric/3860471

**Published:** 2026-09-14

**Authors:** Hadeel Ayesh, Mohammad Al-Shaer, Shoukri Alsleibi

**Affiliations:** ^1^ Department of Cardiology, Bethlehem Arab Society for Rehabilitation, Bethlehem, State of Palestine

**Keywords:** angiography, case report, culprit, emergent, nondominant, right coronary artery, ST-segment elevation myocardial infarction

## Abstract

Acute occlusion of a nondominant right coronary artery (RCA) is an uncommon but clinically significant cause of anterior ST‐segment elevation that may mimic anterior ST‐segment elevation myocardial infarction (STEMI) and pose a diagnostic challenge in identifying the culprit vessel during emergent coronary angiography. We report two patients presenting with anterior ST‐segment elevation in whom emergent coronary angiography revealed acute occlusion of a nondominant RCA with right ventricular branch compromise. In both cases, chest pain and ST‐segment elevation improved immediately after RCA reperfusion and before intervention on other coronary lesions. These cases highlight the importance of recognizing this atypical presentation and carefully assessing coronary anatomy to identify the culprit vessel and guide timely revascularization.

## 1. Introduction

Right ventricular myocardial infarction (RVMI) most commonly occurs in association with inferior wall myocardial infarction and is typically caused by proximal occlusion of a dominant right coronary artery (RCA) [1]. In contrast, isolated RVMI due to occlusion of a nondominant right coronary artery (NDRCA) is uncommon, accounting for less than 3% of myocardial infarctions in autopsy series and remains infrequently recognized in clinical practice [2]. Its diagnosis is often challenging because clinical and electrocardiographic manifestations may be atypical and misleading.

Coronary dominance is defined by the artery giving rise to the posterior descending artery (PDA). Approximately 80% of individuals have a right‐dominant circulation, 10% are left‐dominant, and 10% are codominant. In left‐dominant coronary circulation, the PDA arises from the left circumflex artery, rendering the NDRCA with a comparatively limited myocardial territory [3–5]. In this setting, the RCA primarily supplies right‐sided cardiac structures, including the right atrium, right ventricular free wall, and right ventricular outflow tract via the conus and acute marginal branches, and frequently gives rise to the sinoatrial nodal branch [4, 5]. Owing to the absence of inferior left ventricular supply, the NDRCA is typically smaller in caliber and confined to right‐sided cardiac structures. Occlusion of a NDRCA is therefore often presumed to be clinically benign; however, occlusion may result in potentially serious clinical consequences [6–8].

Electrocardiographically, RVMI is classically diagnosed by ST‐segment elevation in right‐sided precordial leads, particularly V4R [9, 10]. In isolated RVMI, especially in left‐dominant coronary systems, the absence of opposing inferior or posterior left ventricular injury currents may allow right ventricular injury currents to manifest as ST‐segment elevation in the anterior precordial leads [11–13]. This rare presentation may closely mimic anterior ST‐segment elevation myocardial infarction (STEMI) and lead to diagnostic uncertainty, delayed recognition, or misidentification of the culprit vessel, particularly in the setting of multivessel coronary artery disease.

We report two cases of acute occlusion of a NDRCA presenting with anterior ST‐segment elevation, highlighting this uncommon electrocardiographic pattern, the diagnostic challenges, and important considerations regarding coronary intervention strategy.

## 2. Case 1

A 71‐year‐old man presented with a 6‐h history of retrosternal chest pain described as burning in nature, radiating to the left arm and associated with diaphoresis. His medical history included Type 2 diabetes mellitus and hypertension for 15 years, active smoking, and prior percutaneous coronary intervention (PCI) to the left anterior descending artery (LAD) 5 years earlier. His medications included vildagliptin/metformin, aspirin, bisoprolol, amlodipine, rosuvastatin, and omeprazole.

**Figure 1:**
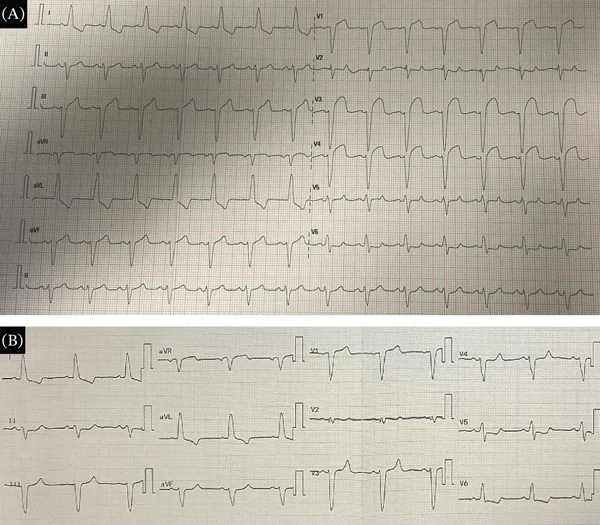
(A) Admission electrocardiogram demonstrating left bundle branch block (LBBB), with approximately 2 mm of discordant ST‐segment elevation in V1, 1 mm of concordant ST‐segment depression in V2, and 3 mm of discordant ST‐segment elevation in V3‐V4. (B) Post‐PCI electrocardiogram demonstrating resolution of the ST‐segment abnormalities in V2–V4, with approximately 1 mm of residual ST‐segment elevation in V1.

On examination, the patient was in distress due to chest pain. Blood pressure was 160/90 mmHg, and oxygen saturation was 95% on room air. Cardiac examination revealed a regular rhythm without murmurs, and lung fields were clear. The admission electrocardiogram demonstrated sinus rhythm with left bundle branch block (LBBB), with approximately 2 mm of discordant ST‐segment elevation in V1, 1 mm of concordant ST‐segment depression in V2, and 3 mm of discordant ST‐segment elevation in V3 and V4. The ST/S ratios in V1, V3, and V4 were 17%, 18%, and 20%, respectively; therefore, the modified Sgarbossa criterion for excessive discordant ST‐segment elevation was not met. The 1‐mm concordant ST‐segment depression in V2 fulfilled the concordant ST‐depression criterion of the original and modified Sgarbossa criteria and the concordant ST‐deviation criterion of the BARCELONA algorithm [14–16]. Nevertheless, the convex morphology of the ST‐segment elevation in V3–V4 was considered an additional concerning feature (Figure 1A). No previous ECG was available for comparison; therefore, the chronicity of the LBBB could not be established.

**Figure 2:**
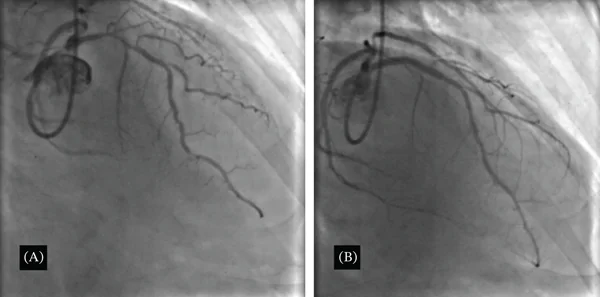
(A) Pre‐PCI left coronary angiogram in the right anterior oblique (RAO) cranial view and (B) post‐PCI angiogram following left anterior descending (LAD) artery intervention, showing a satisfactory angiographic result.

**Figure 3:**
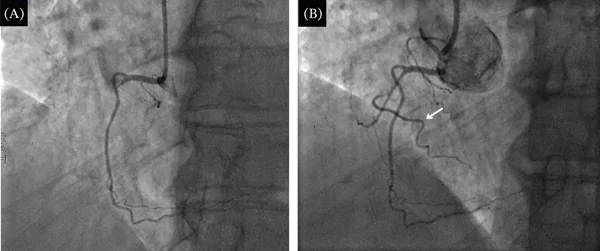
Pre‐PCI right coronary artery (RCA) angiogram of a (A) nondominant RCA and (B) post‐PCI angiogram demonstrating successful revascularization of the right ventricular branch (white arrow).

Given concern for acute anterior STEMI, emergent coronary angiography was performed. The left main coronary artery was free of significant disease. The LAD demonstrated a patent proximal stent with significant instent restenosis extending beyond the stent margins, as well as a severe mid/distal focal lesion (Figure 2A); however, antegrade flow was preserved with TIMI 3 flow throughout the LAD, and no angiographic features of acute thrombotic occlusion were observed. The left circumflex artery was dominant with nonobstructive disease. In contrast, the RCA was nondominant and demonstrated acute mid‐segment total occlusion with TIMI 0 flow, involving the right ventricular branch (Figure 3A).

The NDRCA was treated first. A workhorse guidewire was advanced across the lesion without the need for specialized crossing techniques, followed by multiple balloon inflations in the mid segment. Flow was restored to the RCA and its compromised right ventricular branch, with final TIMI 2 flow and no angiographic dissection. Given the small vessel caliber, balloon angioplasty alone was considered preferable to stent implantation, and the final angiographic result was considered acceptable (Figure 3B). Importantly, the patient′s chest pain and the anterior ST‐segment elevation observed on the monitor resolved immediately after RCA reperfusion, before intervention on the LAD.

Although visible thrombus was not observed in this lesion, the abrupt occlusion, complete loss of flow, involvement of the right ventricular branch, and subsequent immediate clinical and electrocardiographic response to RCA reperfusion supported the NDRCA as the culprit vessel.

Following reperfusion of the presumed culprit vessel, the decision was made to proceed with PCI of the LAD because of the presence of severe disease in a major prognostically important artery. Predilatation of the mid/distal LAD lesion was performed, followed by implantation of a second‐generation drug‐eluting stent with an excellent angiographic result. Then, high‐pressure noncompliant balloon dilatation of the proximal LAD instent restenosis was undertaken, followed by implantation of an additional second‐generation drug‐eluting stent extending beyond the prior stent margins, with subsequent high‐pressure postdilatation and an excellent final angiographic result in both treated segments (Figure 2B).

Drug‐eluting stent implantation was selected over drug‐coated balloon angioplasty for the proximal LAD instent restenosis because the restenosis disease extended beyond the original stent margins. Given the emergent nature of the procedure, the angiographically evident restenosis, and the satisfactory final angiographic result, intravascular imaging was not performed, although it could have provided additional information regarding the mechanism of restenosis and stent optimization. The absence of intravascular imaging is acknowledged as a limitation.

Postprocedural electrocardiography demonstrated persistent LBBB, with resolution of the ST‐segment abnormalities in V2–V4 and approximately 1 mm of residual ST‐segment elevation in V1 (Figure 1B). The patient remained chest pain–free and hemodynamically stable during his stay in the coronary care unit.

Laboratory testing revealed a total cholesterol level of 233 mg/dL, LDL cholesterol of 132 mg/dL, triglycerides of 295 mg/dL, and hemoglobin A1c of 5.6%. Admission troponin I was 0.1 ng/mL, rising to 2.2 ng/mL at 6 h and peaking at 6.7 ng/mL. Transthoracic echocardiography demonstrated mildly reduced left ventricular systolic function without regional wall‐motion abnormalities, moderate aortic regurgitation, and no pericardial effusion. Detailed quantitative assessment of right ventricular function, including TAPSE, tricuspid annular S ^′^ velocity, fractional area change, and regional right ventricular wall motion, was not performed. Cardiac magnetic resonance imaging was not performed.

The patient was discharged in stable condition on aspirin 100 mg once daily, ticagrelor 90 mg twice daily, rosuvastatin 20 mg once daily, dapagliflozin 10 mg once daily, valsartan/amlodipine 80/5 mg once daily, bisoprolol 2.5 mg once daily, and pantoprazole 40 mg once daily. He remained asymptomatic during 1 year of outpatient follow‐up.

## 3. Case 2

A 58‐year‐old man with a history of chronic obstructive pulmonary disease and active smoking presented with a 4‐h history of severe retrosternal chest pain described as heavy in nature, associated with dyspnea and diaphoresis. On examination, he appeared uncomfortable. Blood pressure was 140/90 mmHg. Lung examination revealed bilaterally diminished air entry, whereas cardiac examination revealed a regular rhythm without murmurs.

**Figure 4:**
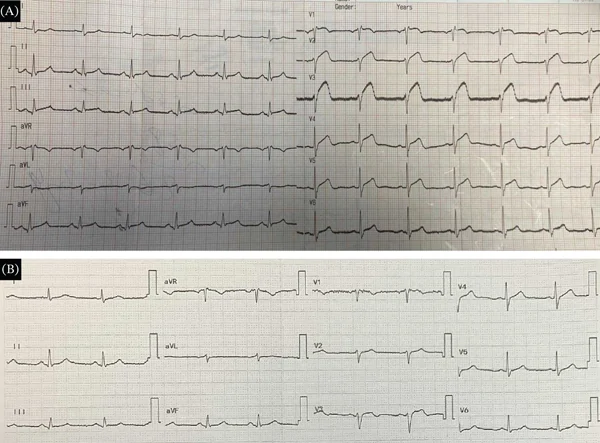
(A) Admission electrocardiogram demonstrating prominent anterior precordial ST‐segment elevation, with mild inferior ST‐segment elevation, and (B) post‐PCI electrocardiogram showing resolution of ST‐segment elevation.

The admission electrocardiogram demonstrated sinus rhythm with approximately 1 mm of ST‐segment elevation in the inferior leads, in addition to more prominent ST‐segment elevation in the anterior precordial leads, particularly V2–V3, where the ST‐segment elevation had a convex morphology (Figure 4A). Given the prominent anterior ST‐segment elevation and clinical presentation, emergency coronary angiography was performed.

**Figure 5:**
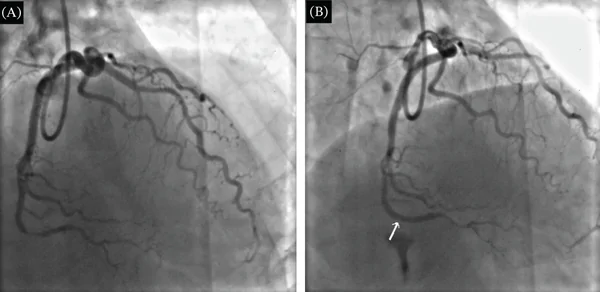
(A) Pre‐PCI left coronary angiogram in the right anterior oblique (RAO) cranial view demonstrating mild to moderate mid–left anterior descending (LAD) artery stenosis and significant left posterior descending artery (L‐PDA) stenosis, and (B) post–L‐PDA PCI angiogram showing a satisfactory angiographic result (white arrow).

**Figure 6:**
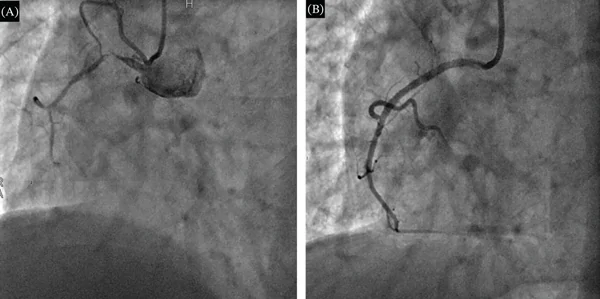
(A) Pre‐PCI right coronary artery (RCA) angiogram demonstrating thrombotic subtotal occlusion of the proximal nondominant RCA, and (B) post‐PCI angiogram showing restoration of coronary flow.

The left main coronary artery was normal. The LAD demonstrated mild‐to‐moderate mid‐segment disease with preserved antegrade flow. The left circumflex artery was dominant, with moderate disease involving the proximal obtuse marginal branch. The left PDA exhibited severe focal stenosis (Figure 5A). The RCA was nondominant and demonstrated proximal subtotal occlusion with angiographic thrombus and delayed antegrade flow, with compromise of the right ventricular branch (Figure 6A).

The NDRCA was treated with balloon predilatation followed by implantation of a second‐generation drug‐eluting stent extending from the proximal to the mid segment. Poststent angiography demonstrated a no‐reflow with TIMI 0 flow. Initial proximal intracoronary administration of one dose of 100 *μ*g of nitroglycerin and three doses of 80‐*μ*g adenosine resulted in limited improvement. Distal intracoronary delivery via a microcatheter was then performed, using two 100‐*μ*g doses of nitroglycerin and three 80‐*μ*g doses of adenosine, with subsequent improvement to TIMI 2 antegrade flow (Figure 6B). A glycoprotein IIb/IIIa inhibitor was not administered because the final angiographic result was considered satisfactory and the anticipated incremental benefit was judged insufficient to justify the additional bleeding risk.

The patient′s chest pain and ST‐segment elevation resolved immediately after RCA reperfusion and before intervention on the left PDA.

The presence of angiographically visible thrombus with delayed antegrade flow, with compromise of the right ventricular branch, together with the subsequent clinical and electrocardiographic response to RCA reperfusion, supported the NDRCA as the culprit vessel.

Percutaneous intervention of the left PDA was subsequently performed with balloon predilatation followed by implantation of a second‐generation drug‐eluting stent, achieving an excellent angiographic result (Figure 5B).

The patient′s chest pain remained resolved, and repeat electrocardiography demonstrated resolution of the anterior ST‐segment elevation, with normalization of the mild inferior ST‐segment elevation (Figure 4B). Transthoracic echocardiography revealed preserved left ventricular systolic function, mild mitral regurgitation, and no pericardial effusion. Right‐sided precordial leads were not obtained before angiography because the initial clinical and electrocardiographic presentation was interpreted as anterior STEMI, prompting direct emergent coronary angiography. Postreperfusion right‐sided ECG assessment would have had limited diagnostic value for establishing the original culprit territory. Detailed quantitative assessment of right ventricular function was not performed. Cardiac magnetic resonance imaging was not performed.

Troponin I level was 0.54 ng/mL on admission, rose to a peak of 70 ng/mL, and decreased to 13.6 ng/mL at discharge.

The patient remained clinically stable and was discharged after 3 days on aspirin 100 mg once daily, ticagrelor 90 mg twice daily, rosuvastatin 20 mg once daily, valsartan 80 mg once daily, and pantoprazole 40 mg once daily. At 1‐year outpatient follow‐up, he remained asymptomatic without recurrent chest pain.

## 4. Discussion

These cases illustrate an uncommon but clinically important presentation of acute NDRCA occlusion manifesting as anterior ST‐segment elevation. Although RVMI accompanies inferior myocardial infarction in up to 25%–50% of cases, isolated RVMI remains uncommon and is frequently underrecognized in clinical practice [13]. In both cases, the clinical and angiographic findings support RVMI as the likely mechanism underlying the anterior ST‐segment elevation. In both cases, the NDRCA was acutely occluded with compromise of the RV branch, and restoration of flow was followed by resolution of the anterior ST‐segment elevation. This pattern is consistent with previously reported cases of RV infarction associated with NDRCA occlusion [11–13]. Accordingly, the diagnosis of RVMI was based on the coronary anatomy, RV branch compromise, ECG pattern, and response to reperfusion, rather than on right‐sided ECG leads, CMR, or detailed quantitative echocardiographic assessment. The absence of CMR means the extent of RV myocardial necrosis was not independently characterized.

In the first case, diagnostic complexity was compounded by the presence of LBBB, a well‐recognized limitation to conventional electrocardiographic interpretation in acute myocardial infarction [14]. No previous ECG was available; therefore, the chronicity of the LBBB could not be established. The admission ECG demonstrated approximately 2 mm of discordant ST‐segment elevation in V1, 1 mm of concordant ST‐segment depression in V2, and 3 mm of discordant ST‐segment elevation in V3–V4. The concordant ST‐segment depression in V2 fulfilled the concordant ST‐depression criterion of the original Sgarbossa criteria and the corresponding criterion retained in the modified Sgarbossa criteria [14, 15]. It also fulfilled the concordant ST‐deviation criterion of the BARCELONA algorithm proposed by Di Marco et al. [16], which considers ≥ 1 mm of concordant ST‐segment deviation in any lead a positive criterion. The discordant ST‐segment elevation in V3–V4 did not meet the modified Sgarbossa criterion for excessive discordant ST‐segment elevation, defined as ST‐segment elevation ≥ 25% of the preceding S‐wave amplitude [15]. Nevertheless, the convex morphology of the ST‐segment elevation in V3–V4 was considered a concerning feature in the clinical context. The precordial distribution, characterized by ST‐segment elevation in V1 and V3–V4 with concordant ST‐segment depression in V2, has been described in association with right ventricular infarction and may support right ventricular involvement [17]. However, these findings are not specific for right ventricular infarction and require cautious interpretation in the presence of LBBB. In this case, the angiographic demonstration of acute NDRCA occlusion with right ventricular branch compromise provided an anatomical substrate for right ventricular ischemia. The subsequent resolution of the ST‐segment abnormalities in V2–V4 following successful RCA reperfusion provided additional support for the ischemic nature of these ECG abnormalities, although approximately 1 mm of ST‐segment elevation persisted in V1. The ECG therefore prompted emergent angiography, which identified two potentially competing abnormalities: severe LAD disease and an acutely occluded NDRCA.

The severe LAD disease initially raised concern that the anterior ST‐segment elevation might be attributable to LAD ischemia. However, several findings favored the NDRCA as the presumed culprit. The LAD had preserved TIMI 3 flow, no angiographic evidence of acute thrombotic occlusion, and no new regional left ventricular wall‐motion abnormality. In contrast, the RCA had an abrupt mid‐segment total occlusion with TIMI 0 flow and right ventricular branch compromise. Although visible thrombus was not demonstrated in Case 1, the abrupt occlusion and subsequent restoration of right ventricular branch flow were temporally associated with immediate resolution of chest pain and ST‐segment elevation before any LAD intervention. The uncomplicated passage of a workhorse guidewire across the lesion was not used as proof of thrombotic acuity; rather, it was considered a procedural observation within the broader clinical and angiographic context.

The second case provided additional evidence because the NDRCA showed angiographically visible thrombus, proximal subtotal occlusion, delayed antegrade flow, and right ventricular branch compromise. The occurrence of no‐reflow after stenting was consistent with distal embolization and microvascular dysfunction in the setting of an acute thrombotic lesion. As in Case 1, the chest pain and ST‐segment elevation resolved following reperfusion of the NDRCA and before intervention on the left PDA. Notably, no intervention was performed on the LAD in this case, as it demonstrated only mild‐to‐moderate disease with preserved antegrade flow, further arguing against an LAD culprit lesion as the cause of the anterior ST‐segment elevation. The close temporal relationship between RCA reperfusion and resolution of both the chest pain and ST‐segment elevation in both cases strengthens the attribution of the ECG abnormalities to acute ischemia in the right ventricular territory.

In Case 2, mild ST‐segment elevation was also present in the inferior leads on the admission ECG. The combination of anterior and inferior ST‐segment elevation may occur with acute occlusion of a wrap‐around LAD, in which the LAD extends beyond the apex to supply part of the inferior wall [18]. In this case, however, coronary angiography demonstrated that the LAD did not have a wrap‐around course and showed only mild‐to‐moderate mid‐segment disease with preserved antegrade flow. This finding illustrates that the presence of concomitant anterior and inferior ST‐segment elevation does not necessarily indicate an LAD‐related infarction and that the ECG pattern should be interpreted in conjunction with the underlying coronary anatomy and angiographic findings.

The observed electrocardiographic pattern can be explained by coronary dominance. In left‐dominant systems, the inferior left ventricular wall is supplied by the circumflex artery and spared during NDRCA occlusion. In the absence of opposing inferior or posterior left ventricular injury currents, right ventricular injury currents may manifest in the anterior precordial leads [11–13], mimicking anterior STEMI and potentially leading to diagnostic delay or misdirected intervention.

Although occlusion of a NDRCA is often presumed to be benign, this vessel supplies critical structures including the right ventricular free wall, right ventricular outflow tract, right atrium, and sinoatrial node. Compromise of right ventricular branches, as seen in both cases, may result in significant ischemic burden despite small vessel caliber. Ischemia in these regions has been associated with ventricular and atrial arrhythmias, hemodynamic compromise, and, in rare cases, sudden cardiac death [6–8]. These considerations underscore the importance of early recognition and prompt revascularization.

Regarding interventional strategy, in Case 1, balloon angioplasty alone was selected because of the small vessel caliber and the absence of dissection, with final TIMI 2 flow and an acceptable angiographic result.

In both cases, significant nonculprit coronary lesions were treated during the same procedure after restoration of RCA flow. This approach was undertaken in the setting of hemodynamic stability, severe disease involving major epicardial vessels, and the clinical judgment that complete revascularization during the index procedure was appropriate. Current evidence supports complete revascularization in appropriate hemodynamically stable patients with STEMI and multivessel coronary artery disease, whereas the timing of nonculprit PCI may be individualized according to the clinical and angiographic context. In the COMPLETE trial, complete revascularization reduced the risk of cardiovascular death or myocardial infarction compared with culprit‐lesion‐only PCI, with the benefit observed regardless of the intended timing of nonculprit PCI [19]. The 2023 ESC Guidelines for acute coronary syndromes also support complete revascularization in appropriate patients with STEMI and multivessel disease [20].

Drug‐eluting stent implantation was selected over drug‐coated balloon angioplasty for LAD instent restenosis in the first case because the restenosis extended beyond stent margins. Intravascular imaging was not performed during LAD treatment. IVUS could have provided useful information regarding the mechanism of instent restenosis and stent optimization, but the procedure was performed emergently and satisfactory angiographic results were obtained. This limitation is acknowledged.

In the second case, no‐reflow after RCA stenting was likely related to thrombus burden, distal embolization, and microvascular obstruction. Angiographically visible thrombus provided direct evidence of an acute thrombotic lesion, and the subsequent no‐reflow phenomenon was consistent with distal embolization and microvascular dysfunction, further supporting the NDRCA as the culprit lesion. Distal intracoronary delivery of adenosine and nitroglycerin through a microcatheter was followed by improvement from TIMI 0 to TIMI 2 flow. A glycoprotein IIb/IIIa inhibitor was not administered because flow improved after distal drug delivery and the anticipated incremental benefit was judged insufficient to justify the additional bleeding risk. The explanation that distal delivery improved drug effect by achieving greater local exposure at the site of distal microvascular dysfunction is plausible but remains inferential in this case. The extensive thrombotic burden and microvascular involvement provide a plausible explanation for the markedly higher troponin elevation observed in this patient compared with the first case, despite preserved left ventricular systolic function.

Several limitations should be acknowledged. Right‐sided precordial leads were not obtained before reperfusion, detailed quantitative right ventricular echocardiographic assessment was not performed, and cardiac magnetic resonance imaging was not performed to independently characterize right ventricular myocardial edema or necrosis. In addition, intravascular imaging was not performed during treatment of the LAD instent restenosis in Case 1.

## 5. Conclusion

Acute NDRCA occlusion is a rare but clinically important differential diagnosis in patients presenting with anterior ST‐segment elevation, particularly in the setting of left‐dominant coronary circulation. This presentation may mimic anterior STEMI and can complicate identification of the culprit vessel, especially when concomitant coronary artery disease is present. Careful assessment of coronary dominance, angiographic evaluation of the right ventricular branches, and correlation of the ECG findings with coronary anatomy are important for accurate culprit‐vessel identification and timely revascularization. A practical framework summarizing these key differentiating features is presented in Table 1.

**Table 1:** Practical differential diagnosis of anterior ST‐segment elevation in acute coronary occlusion.

Feature	Classic anterior LAD STEMI	Nondominant RCA occlusion with RV involvement	Wrap‐around LAD occlusion
Predominant myocardial territory	Anterior LV ± septal/apical territory	Right ventricular/right‐sided territory	Anterior/apical ± inferior LV territory
Typical angiographic finding	Acute LAD occlusion	Acute NDRCA occlusion with RV branch compromise	LAD occlusion with a wrap‐around course
Anterior ST elevation	Common	Can occur	Common
Inferior ST elevation	Usually absent, but may occur	Usually absent; may vary with anatomy	May occur
Right‐sided ECG leads	Usually not diagnostic	V4R may support RV involvement	Usually not diagnostic
Key discriminator	Acute LAD occlusion corresponding to the ECG territory	Acute NDRCA occlusion with RV branch compromise	Wrap‐around LAD anatomy with apical/inferior LV involvement

*Note:* This table is an author‐constructed practical synthesis based on published literature [9–13, 17, 18] and is not intended as a validated diagnostic algorithm.

**Nomenclature**
CCU: coronary care unit
ECG: electrocardiogram
LBBB: left bundle branch block
LCX: left circumflex artery
NDRCA: nondominant right coronary artery
PCI: percutaneous coronary intervention
PDA: posterior descending artery
RCA: right coronary artery
RVMI: right ventricular myocardial infarction
STEMI: ST‐segment elevation myocardial infarction

## Funding

No funding was received for this manuscript.

## Consent

Written informed consent was obtained from both patients for publication of this case report and any accompanying images.

## Conflicts of Interest

The authors declare no conflicts of interest.

## Data Availability

The data that support the findings of this study are available on request from the corresponding author. The data are not publicly available due to privacy or ethical restrictions.
